# Supplementary material for: Development of a computer-aided design software for the quantitative evaluation of aesthetic damage
Source: PLoS One. 2019 Dec 18;14(12):e0226322. doi: 10.1371/journal.pone.0226322 (PMC6919621; doi:10.1371/journal.pone.0226322)
Supplement: S1 Code site — 1. (ZIP) [file pone.0226322.s001.zip › [EESC_jr][Projeto]Relatorio_07_21/reports/templates_zip/gentelella-master/vendors/Flot/examples/percentiles/index.html]

Flot Examples: Percentiles


## Percentiles

Height in centimeters of individuals from the US (2003-2006) as function of age in years (source: CDC). The 15%-85%, 25%-75% and 50% percentiles are indicated.

For each point of a filled curve, you can specify an arbitrary bottom. As this example illustrates, this can be useful for plotting percentiles. If you have the data sets available without appropriate fill bottoms, you can use the fillbetween plugin to compute the data point bottoms automatically.

Copyright © 2007 - 2014 IOLA and Ole Laursen
